# Low Rates of Both Lipid-Lowering Therapy Use and Achievement of Low-Density Lipoprotein Cholesterol Targets in Individuals at High-Risk for Cardiovascular Disease across Europe

**DOI:** 10.1371/journal.pone.0115270

**Published:** 2015-02-18

**Authors:** Julian P. Halcox, Florence Tubach, Esther Lopez-Garcia, Guy De Backer, Claudio Borghi, Jean Dallongeville, Eliseo Guallar, Jesús Medina, Joep Perk, Ogün Sazova, Stephen Sweet, Carine Roy, José R. Banegas, Fernando Rodriguez-Artalejo

**Affiliations:** 1 Institute of Life Sciences 2, Swansea University College of Medicine, Swansea, United Kingdom; 2 INSERM CIC-EC 1425 and Epidemiology and Clinical Research Department, Assistance Publique—Hôpitaux de Paris, Hôpital Bichat, Paris, France; 3 Univ Paris Diderot, Sorbonne Paris Cité, UMR 1123, Paris, France; 4 Department of Preventative Medicine and Public Health, Universidad Autónoma de Madrid IdiPaz and CIBERESP, Madrid, Spain; 5 Department of Public Health, University of Ghent, Ghent, Belgium; 6 Department of Internal Medicine, Ageing and Clinical Nephrology, University of Bologna, Bologna, Italy; 7 INSERM U 744, Institut Pasteur de Lille, Université Lille-Nord de France, Lille, France; 8 Departments of Epidemiology and Medicine and Welch Center of Prevention, Epidemiology and Clinical Research, Johns Hopkins Bloomberg School of Public Health, Baltimore, Maryland, United States of America; 9 Department of Cardiovascular Epidemiology and Population Genetics, National Center for Cardiovascular Research, Madrid, Spain; 10 Observational Research Centre, Global Medical Affairs, AstraZeneca Farmacéutica Spain, Madrid, Spain; 11 School of Health and Caring Sciences, Linnaeus University, Kalmar, Sweden; 12 AstraZeneca Global Medical Affairs, London, United Kingdom; 13 Research Evaluation Unit, Oxford PharmaGenesis Ltd, Oxford, United Kingdom; UMR INSERM U866, FRANCE

## Abstract

**Aims:**

To analyse the treatment and control of dyslipidaemia in patients at high and very high cardiovascular risk being treated for the primary prevention of cardiovascular disease (CVD) in Europe.

**Methods and Results:**

Data were assessed from the European Study on Cardiovascular Risk Prevention and Management in Usual Daily Practice (EURIKA, ClinicalTrials.gov identifier: NCT00882336), which included a randomly sampled population of primary CVD prevention patients from 12 European countries (*n* = 7641). Patients’ 10-year risk of CVD-related mortality was calculated using the Systematic Coronary Risk Evaluation (SCORE) algorithm, identifying 5019 patients at high cardiovascular risk (SCORE ≥5% and/or receiving lipid-lowering therapy), and 2970 patients at very high cardiovascular risk (SCORE ≥10% or with diabetes mellitus). Among high-risk individuals, 65.3% were receiving lipid-lowering therapy, and 61.3% of treated patients had uncontrolled low-density lipoprotein cholesterol (LDL-C) levels (≥2.5 mmol/L). For very-high-risk patients (uncontrolled LDL-C levels defined as ≥1.8 mmol/L) these figures were 49.5% and 82.9%, respectively. Excess 10-year risk of CVD-related mortality (according to SCORE) attributable to lack of control of dyslipidaemia was estimated to be 0.72% and 1.61% among high-risk and very-high-risk patients, respectively. Among high-risk individuals with uncontrolled LDL-C levels, only 8.7% were receiving a high-intensity statin (atorvastatin ≥40 mg/day or rosuvastatin ≥20 mg/day). Among very-high-risk patients, this figure was 8.4%.

**Conclusions:**

There is a considerable opportunity for improvement in rates of lipid-lowering therapy use and achievement of lipid-level targets in high-risk and very-high-risk patients being treated for primary CVD prevention in Europe.

## Introduction

Elevated serum total cholesterol and low-density lipoprotein cholesterol (LDL-C) levels are among the primary causal risk factors for cardiovascular disease (CVD) [1, 2, 3]. We have previously shown in the European Study on Cardiovascular Risk Prevention and Management in Usual Daily Practice (EURIKA; ClinicalTrials.gov identifier: NCT00882336) that dyslipidaemia remains prevalent in Europe, with a total of 57.7% of patients with at least one major risk factor for CVD but no history of cardiovascular events (a primary CVD prevention population) being dyslipidaemic [4]. Among the general population in Europe, prevalence estimates for dyslipidaemia range between approximately 30% and 60% [5, 6, 7, 8, 9, 10].

The European guidelines on CVD prevention recommend adapting strategies for the primary prevention of CVD in accordance with patients’ overall level of cardiovascular risk [3]. According to these guidelines, global cardiovascular risk in individual patients should be assessed using the Systematic Coronary Risk Evaluation (SCORE) algorithm, which estimates patients’ 10-year risk of death due to CVD based on their age, sex, smoking status, blood pressure, and lipid levels [1, 3, 11, 12]. Patients are considered to be at high cardiovascular risk if they have a SCORE of ≥5 to <10%, type 1 or 2 diabetes mellitus with no other cardiovascular risk factors, or moderate chronic kidney disease. Patients are considered to be at very-high-risk if they have a SCORE of ≥10%, a history of CVD, type 1 or 2 diabetes mellitus with other cardiovascular risk factors or target organ damage, or severe chronic kidney disease [3].

Recommended target levels for total cholesterol are <5 mmol/L for all individuals, and recommended target levels for LDL-C are <3 mmol/L for those at intermediate cardiovascular risk, <2.5 mmol/L for those at high-risk and <1.8 mmol/L or at least a 50% reduction for those at very-high-risk. For individuals exceeding these recommended levels, lifestyle advice is recommended for those at intermediate-risk, with drug treatment to be considered if levels remain uncontrolled, and by early drug intervention for those at high-risk or very-high-risk [3]. Statins are recommended as the therapy of first choice in patients requiring pharmacological intervention to reduce total cholesterol and LDL-C levels [3]. Most guidelines suggest that statin therapy should be considered to reduce global CVD risk in patients at high-risk and very-high-risk even if total cholesterol and LDL-C concentrations are at or below target levels [12, 13]. The recent ACC and AHA guidelines in the USA no longer recommend a target based approach to the use of lipid lowering therapy. High or moderate intensity statin therapy is recommended according to the risk status of the individual patient. In contrast, the European guidelines continue to recommend a target-based approach [14, 15].

As a further analysis of the data from EURIKA, we have assessed how dyslipidaemia is treated throughout Europe, specifically exploring how well patients’ cholesterol levels are controlled and what potential evidence-based improvements in therapy could be considered for those not at guideline-recommended target cholesterol levels.

## Methods

### Study design and participants

EURIKA was carried out in 12 European countries (Austria, Belgium, France, Germany, Greece, Norway, Russia, Spain, Sweden, Switzerland, Turkey, and the UK) [4]. Data collection started in May 2009 and ended in January 2010, with a 3-month data collection period for each country. The study protocol was approved by the appropriate clinical research ethics committees in each participating country, and all patients provided signed informed consent.

The methods for the study have been reported in detail elsewhere [16]. Briefly, the study sample was selected in a two-stage process that involved the random selection of both physicians and their patients [16, 17]. In the first stage, primary care practitioners (PCPs) and specialists involved in CVD prevention (including cardiologists, endocrinologists, and internal medicine specialists) were randomly selected for invitation to participate using the OneKey database (Cegedim Dendrite, Boulogne-Billancourt, France) [18]. In total, 809 physicians (approximately 60 per country) agreed to participate in EURIKA, 64% of whom were PCPs [17]. Other physician specialties included cardiology, internal medicine and endocrinology. In the second stage, participating physicians invited patients who met the selection criteria (age 50 years or older, free of CVD but having at least one major cardiovascular risk factor [dyslipidaemia, hypertension, smoking, diabetes mellitus, or obesity]) [4]. Approximately 600 patients were included in the EURIKA population per country, with a final population size of 7641. For the present analysis, included patients were those at high CVD risk, defined as those with a SCORE of ≥5% and/or who were already receiving lipid-lowering therapy, or very-high CVD risk, defined as those who had a SCORE of ≥10% or diabetes mellitus. The majority of the very-high-risk patients were also analysed as part of the high-risk group.

### Assessment of CVD risk factors

Demographic information and other details of participating patients were gathered from medical records and patient interviews. For each patient, a physical exam was conducted, blood pressure was measured, and a 12-hour fasting blood sample was collected within 1 day of the initial outpatient consultation [16]. Blood pressure measurements were obtained under standardized conditions for each patient, and blood sample analysis was carried out at a central laboratory (BioAnalytical Research Corporation, Ghent, Belgium) with the exception of patients in Russia, for whom laboratory analysis was carried out locally. High-density lipoprotein cholesterol (HDL-C) concentration was measured by a modified enzymatic method, total cholesterol concentration by the CHOD-PAP method, and triglyceride concentration by the GPO-PAP method (all using the Roche Modular P chemistry analyser [Roche Diagnostics, IN, USA]). LDL-C concentration was calculated by the Friedewald formula [19]. Ten-year CVD-related mortality risk for each patient was estimated using the SCORE algorithms for high-risk and low-risk countries, as appropriate [1].

### Lipid-lowering therapy and control of dyslipidaemia

The use of lipid-lowering therapy, including agents and dosing regimens used, was determined for all patients. For statin treatment, therapy was categorized as low-intensity (pravastatin, simvastatin, lovastatin, fluvastatin, atorvastatin <40 mg/day, or rosuvastatin <20 mg/day) or high-intensity (atorvastatin ≥40 mg/day or rosuvastatin ≥20 mg/day). Control of dyslipidaemia was defined as LDL-C levels <2.5 mmol/L in high-risk patients and <1.8 mmol/L in very-high-risk patients (when very-high-risk patients were analysed as part of the high-risk group, the former definition was used).

### CVD-related mortality risk attributable to lack of control of dyslipidaemia

The excess CVD-related mortality risk (attributable risk; AR) and the percentage of CVD-related mortality risk (%AR) that is attributable to the lack of control of dyslipidaemia was calculated among both high-risk and very-high-risk patients. AR was defined as the absolute difference in SCORE-based, CVD-related mortality risk between the total EURIKA population and these high-risk and very-high-risk participants if all of them had controlled dyslipidaemia (i.e. the complementary risks thus calculated would represent the potential improvement in CVD-related mortality that could be obtained by improving control of dyslipidaemia in these individuals). The %AR was calculated as AR divided by the CVD-related mortality risk in the total EURIKA population; it represents the percentage of CVD-related mortality among the total EURIKA population that is due to the lack of dyslipidaemia control in high-risk and very-high-risk patients for whom medical therapy is recommended (thus, it estimates the potential improvement in CVD-related mortality among the EURIKA population that might be achieved by effective control of dyslipidaemia in those most at risk).

Since the SCORE algorithm utilizes total cholesterol levels for calculation of CVD-related mortality risk, we have assumed that the benefit of LDL-C control was approximately equivalent to the benefit of reducing the level of total cholesterol among the uncontrolled patients to the mean total cholesterol level in patients with controlled dyslipidaemia. Statistical analyses were carried out using SAS (V9.2, SAS Institute Inc., Cary, NC, USA).

## Results

### Patient demographics and general characteristics

Of the 7641 patients in the overall EURIKA population, we defined two groups for analysis: (i) a high-risk group comprising all participants who had a SCORE of ≥5% and/or who were already receiving lipid-lowering therapy, and (ii) a very-high-risk group of patients who had either a SCORE of ≥10% or a diagnosis of diabetes mellitus. For the purposes of this analysis, we considered the PCP’s decision to treat those patients already receiving lipid-lowering therapy as a determination that they were at high-risk. A total of 5019 patients were found to be in the high-risk group (65.7% of the total EURIKA population), 2970 in the very-high-risk group (38.9% of the total EURIKA population). Of the high-risk patients, 2465 (49.1%) were also in the very-high-risk group; only 505 patients with diabetes mellitus in the very-high-risk group were not also included in the high-risk group.

Demographic information for these two groups is presented in Table 1. Patients in the high-risk group had a mean age of 66.1 years, there were more men than women (54.6% vs. 45.4%) and 21.3% were smokers. The prevalence of hypertension was 76.5% and a total of 73.4% had a prior diagnosis of dyslipidaemia. The prevalence of diabetes mellitus was 30.7%. Patient characteristics were generally similar in the high-risk and very-high-risk groups. The mean age of very-high-risk patients was 67.2 years, 42.1% were female and 79.9% had hypertension. The proportion of patients who had received a prior diagnosis of dyslipidaemia (62.1%) was lower in the very-high-risk group than in the high-risk group, and the proportion who had diabetes mellitus (68.9%) was higher. If all patients with diabetes mellitus were excluded from the very-high-risk group, the mean SCORE-based 10-year risk of CVD-related mortality was 17.3% (standard deviation: 7.9).

**Table 1:** Baseline patient characteristics.

	**High-risk (SCORE ≥5%, or receiving LLT)**	**Very-high-risk(SCORE ≥10%, or diabetes mellitus)**
	**(*n* = 5019)**	**(*n* = 2970)**
Age, years	66.1 (8.9)	67.2 (9.5)
Female, *n* (%)	2277 (45.4)	1250 (42.1)
Dyslipidaemia, *n* (%)	3683 (73.4)	1844 (62.1)
TC, mmol/L	5.3 (1.2)	5.2 (1.2)
LDL-C, mmol/L	3.1 (1.0)	3.0 (1.0)
HDL-C, mmol/L	1.4 (0.4)	1.4 (0.4)
TG, mmol/L	1.8 (1.4)	1.9 (1.5)
Hypertension, *n* (%)	3839 (76.5)	2372 (79.9)
SBP, mmHg	137.1 (17.0)	139.5 (17.8)
DBP, mmHg	80.8 (10.1)	80.9 (10.4)
BMI, kg/m^2^	28.8 (5.2)	29.6 (5.7)
Current smoker, *n* (%)	1061 (21.3)	580 (19.7)
Diabetes mellitus, *n* (%)	1541 (30.7)	2046 (68.9)

Data are mean (SD) unless otherwise indicated.

Dyslipidaemia: LDL-C levels ≥4.1 mmol/L (160 mg/dL), or HDL-C levels <1.036 mmol/L (40 mg/dL) for men or <1.300 mmol/L (50 mg/dL) for women, or TG levels ≥1.7 mmol/L (150 mg/dL); hypertension: SBP ≥140 mmHg, or DBP ≥90 mmHg.

BMI, body mass index; DBP, diastolic blood pressure; HDL-C, high-density lipoprotein cholesterol, LDL-C, low-density lipoprotein cholesterol; LLT, lipid-lowering therapy; SBP, systolic blood pressure; SCORE, Systematic Coronary Risk Evaluation; TC, total cholesterol; TG, triglycerides.

### Lipid-lowering therapy and control of LDL-C levels

**Figure 1:**
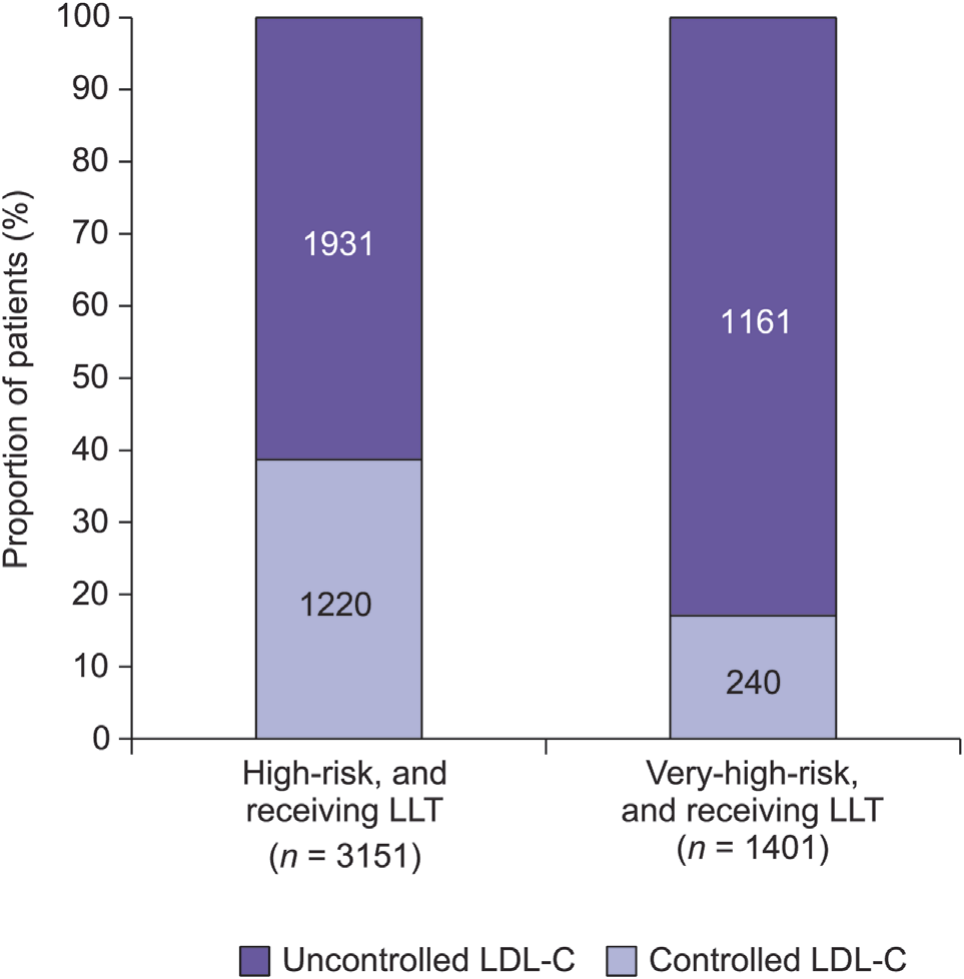
Percentage of patients in the high-risk and very-high-risk groups receiving any form of lipid-lowering therapy (LLT) who have controlled and uncontrolled levels of low-density lipoprotein cholesterol (LDL-C). Patients for whom data regarding LDL-C levels were not available were excluded. Controlled LDL-C levels were defined as <2.5 mmol/L in the high-risk group (including patients who are also included in the very-high-risk group), and <1.8 mmol/L in the very-high-risk group.

**High-risk patients**. Among those in the high-risk group, 3278 (65.3%) were receiving lipid-lowering therapy (Table 2). The majority of these patients (87.3%) were receiving a statin alone. A further 5.4% were receiving a statin in combination with a non-statin lipid-lowering treatment, and 7.3% were receiving a non-statin lipid-lowering treatment alone. Non-statin lipid lowering treatments included ezetimibe, fibrates, nicotinic acid and anion exchange resins. Data concerning LDL-C levels were available for 3151 (96.1%) of those in the high-risk group receiving any form of lipid-lowering therapy, showing that 1931 (61.3%) of these treated patients still had uncontrolled LDL-C levels (≥2.5 mmol/L [mean 3.4]) (Fig. 1). A total of 1231 (39.1%) had LDL-C levels ≥3.0 mmol/L.

**Table 2:** Use of lipid-lowering therapy.

	**High-risk(SCORE ≥5%, or receiving LLT)**	**Very-high-risk(SCORE ≥10%, or diabetes mellitus)**
	**(*n* = 5019)**	**(*n* = 2970)**
Any LLT	3278 (65.3)	1469 (49.5)
Statins alone	2862 (87.3)	1299 (88.4)
Statins with additional non-statin LLT^a^	178 (5.4)	86 (5.9)
Non-statin LLT^a^	238 (7.3)	84 (5.7)

^a^Non-statin LLT: ezetimibe, fibrates, nicotinic acid, anion exchange resins.

LLT, lipid-lowering therapy; SCORE, Systematic Coronary Risk Evaluation.

**Very-high-risk patients**. In total, 1469 (49.5%) patients who had a SCORE of ≥10% or diabetes mellitus were receiving any form of lipid-lowering therapy (Table 2). Among these, 88.4% were receiving a statin alone. A further 5.9% were receiving a statin in combination with a non-statin lipid-lowering treatment, and 5.7% were receiving a non-statin lipid-lowering treatment alone. Data concerning LDL-C levels were available for 1401 (95.4%) of the patients receiving any form of lipid-lowering therapy, of whom 1161 (82.9%) still had uncontrolled LDL-C levels (≥1.8 mmol/L [mean 2.9]) (Fig. 1). A total of 694 (49.5%) had LDL-C levels ≥2.5 mmol/L.

### Attributable risk

Among patients in the high-risk group, the excess SCORE-based 10-year risk of CVD-related mortality attributable to uncontrolled dyslipidaemia (AR) was 0.72% (95% confidence interval [CI]: 0.67–0.77%), which translates into a %AR of 12.1% (95% CI: 11.7–12.6%). For the very-high-risk patients, the corresponding figures were 1.61% (95% CI: 1.49–1.72%) and 16.5% (95% CI: 15.4–16.7%).

### Use of low-intensity and high-intensity statins

**Figure 2:**
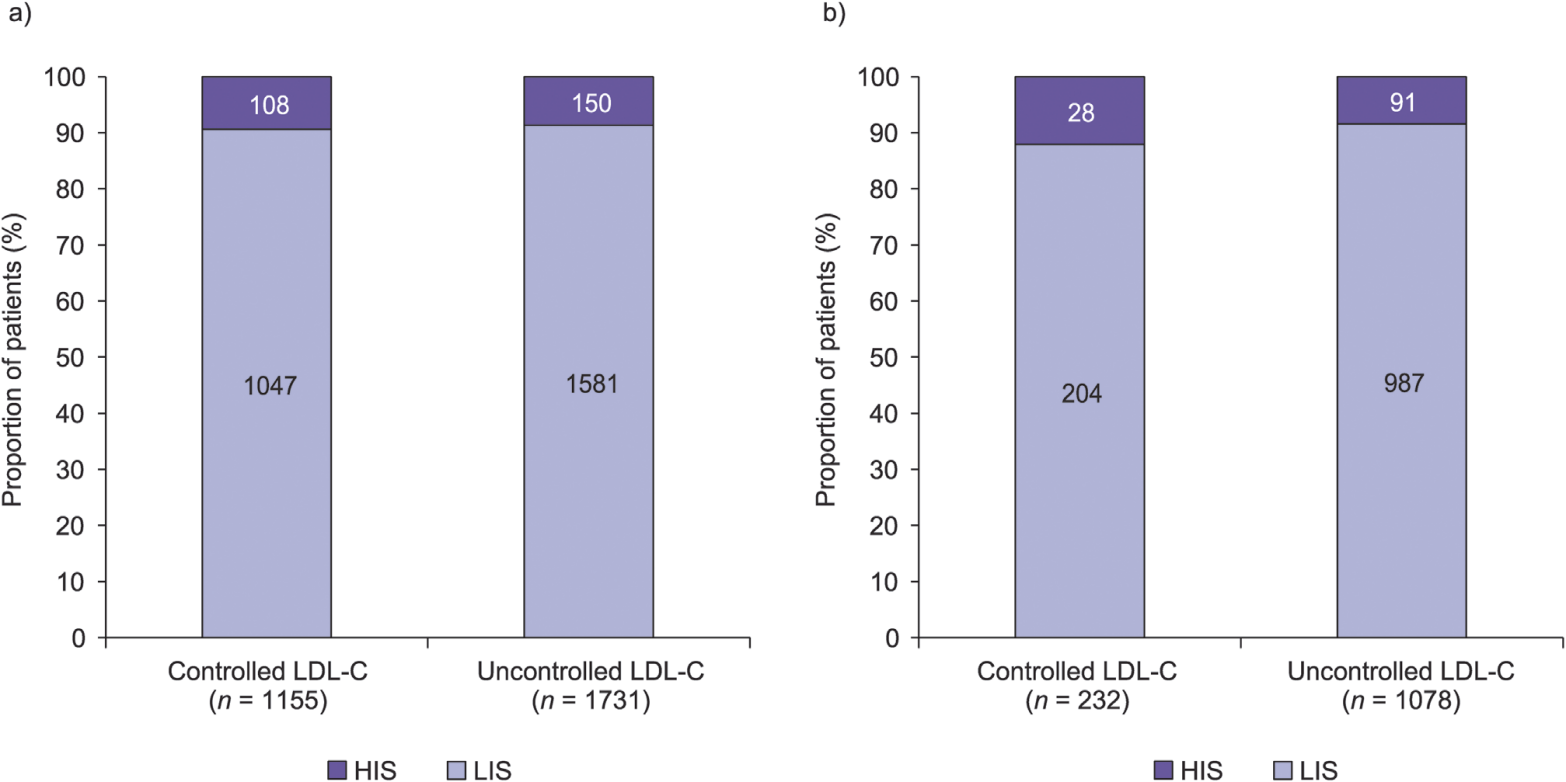
Percentage of high-risk (A) and very-high-risk (B) patients with controlled or uncontrolled low-density lipoprotein cholesterol (LDL-C) levels who are treated with low-intensity statins (LIS) or high-intensity statins (HIS). Controlled LDL-C levels were defined as <2.5 mmol/L in the high-risk group (including patients who are also included in the very-high-risk group), and <1.8 mmol/L in the very-high-risk group. LIS: pravastatin, simvastatin, lovastatin, fluvastatin, atorvastatin <40 mg or rosuvastatin <20 mg; HIS: atorvastatin ≥ 40 mg or rosuvastatin ≥ 20 mg.

The proportions of patients in the high-risk and very-high-risk groups who had controlled or uncontrolled LDL-C levels and who were receiving either a high-intensity or low-intensity statin are shown in Fig. 2. Among the high-risk patients, of those with uncontrolled LDL-C levels only 8.7% were receiving a high-intensity statin. Among the very-high-risk group, of those with uncontrolled LDL-C levels only 8.4% were receiving a high-intensity statin. The proportions of patients receiving high-intensity and low-intensity statins were similar among those with controlled LDL-C levels and those with uncontrolled LDL-C levels in both the high-risk and the very-high-risk groups of patients.

## Discussion

In this analysis of data from a large, multinational study of the primary prevention of CVD in Europe, we have demonstrated that there remains considerable potential for improvement in the treatment of dyslipidaemia. Of the patients in the group defined as being at high-risk of CVD, over a third (34.7%) were not receiving lipid-lowering therapy, despite their elevated level of risk and guidelines recommending pharmacological intervention with a statin in such individuals [12, 13]. A total of 73.4% of patients in this group had received a prior diagnosis of dyslipidaemia. The situation was even more striking in the very-high-risk group, of whom over half (50.5%) were not receiving any form of lipid-lowering therapy, despite their considerable level of risk and a previous diagnosis of dyslipidaemia in almost two thirds (62.1%). Thus, it is likely that many patients in the general population for whom statin therapy would be of benefit are not receiving appropriate treatment.

Furthermore, among those who were receiving any form of lipid-lowering therapy, LDL-C levels were only controlled (according to European guideline recommendations [3]) in 38.7% of the patients in the high-risk group, and 17.1% of those in the very-high-risk group. Approximately 12–16% of the SCORE-based 10-year risk of CVD-related mortality in high-risk and very-high-risk patients is attributable to suboptimal control of dyslipidaemia. Among those with uncontrolled LDL-C levels who were receiving lipid-lowering therapy, only 8.7% and 8.4% of the high-risk and very-high-risk patients, respectively, were receiving a high-intensity statin. Hence, there is considerable potential for optimization of the dose and/or form of statin treatment for high-risk and very-high-risk patients. A further possible explanation for the lack of control of LDL-C levels amont patients receiving lipid lowering therapy is non-compliance to treamtent. Although the data in our study do not allow us to test this hypothesis, it is possible that strategies to improve patient compliance could be of clinical benefit.

It was notable that the proportion of patients receiving a high-intensity statin was similar among patients with controlled dyslipidaemia and patients with uncontrolled dyslipidaemia, in both the high-risk and very-high-risk groups of patients. One potential explanation for this is that PCPs prescribe high-intensity treatment to patients with higher pretreatment cholesterol levels, for whom cholesterol level control is likely to be more difficult to achieve. Unfortunately, the evaluation of pretreatment cholesterol levels was not included in our study, preventing a formal test of this hypothesis. Evidence suggests that patients with uncontrolled LDL-C levels receiving a lower intensity treatment are likely to derive additional benefit from more intensive therapy, if tolerated [3, 12, 13].

We also found that only 5.4% and 5.9% of the high-risk and very-high-risk patients, respectively, were receiving statin therapy in combination with another lipid-lowering agent. There is potential to improve control of lipid levels by addition of other agents to statin therapy, although definitive trial evidence showing that this will result in reductions in CVD event rates over the use of optimal statin doses is currently lacking. The Improved Reduction of Outcomes: Vytorin Efficacy International Trial (IMPROVE-IT), examining whether a fixed-dose combination of ezetimibe and simvastatin offers improved cholesterol-lowering efficiency over simvastatin alone, is still ongoing. Nicotinic acid agents and fibrates may also be considered, although these agents are used more commonly to address HDL-C and triglyceride abnormalities [20, 21].

Our results are in line with those of previous studies: a study conducted in Italy has shown that among patients prescribed a statin, only 22.6% were within 10% of their target LDL-C levels after 12 months of treatment [22]. A literature review published in 2004 has similarly reported a widespread failure to treat patients with dyslipidaemia who should be receiving lipid-lowering therapy, and failure to reach cholesterol target levels among those who are treated [23]. This finding remains true in the USA; a recent systematic review of five population-based studies including data from 18 656 participants found that among patients at high cardiovascular risk, achievement of an LDL-C target of 100 mg/dL increased from only 24% to 50.4% between 1999 and 2008 [24]. In the same time period, the proportion of patients with LDL-C levels ≥ 130 mg/dL and not receiving lipid lowering therapy decreased from 29.4% to 18%. Our study is the first to evaluate these issues systematically across a large group of European patients with varying levels of CVD risk, who have one or more cardiovascular risk factors but who are free from CVD. Previously, we have reported from EURIKA the finding that only 41.2% of patients with dyslipidaemia attained both their total cholesterol and LDL-C target levels [4].

Proper selection of lipid-lowering therapy and effective control of cholesterol levels has been proven to be of benefit: a meta-analysis by the Cholesterol Treatment Trialists’ collaborators has shown that statin treatment can result in a 23% reduction in cardiovascular events and a 19% reduction in CVD-related mortality per 1 mmol/L reduction in patients’ LDL-C levels [25]. The same group has more recently demonstrated that statin treatment is of benefit to patients across the whole spectrum of CVD risk, even in those at low-risk and intermediate-risk [26]. It should be noted, however, that the absolute magnitude of risk reduction with statin therapy is greater with increasing level of risk. Thus, both the clinical- and cost-effectiveness of statin therapy increases with patients’ absolute level of cardiovascular risk. Although a recent retrospective study has shown that patients initiating a high intensity statin have a higher probability of hospitalization due to acute kidney injury (AKI) in the first 3 months of therapy than patients initiating a low-intensity statin therapy [27], the absolute risk of AKI is low. Comparative data regarding the impact of therapy on hard cardiovascular outcomes in the study population was not reported, and the largest meta-analysis of randomised controlled trials to date has failed to detect a significant rate of AKI in patients receiving statins [26]. Therefore, although the exact impact of statin therapy on AKI remains controversial, the potential risk is currently outweighed by the established cardiovascular benefit of effective therapy in the intermediate-risk to high-risk patients so far studied.

A strength of our study was the testing of a large sample of patients from multiple countries according to standardized procedures. The participation acceptance rate among physicians was low, but the random selection of patients and a relatively high patient acceptance rate of 62.1% are likely to have reduced patient selection bias. As the data-collection period for each country was only 3 months, it is possible that frequent healthcare service users were overrepresented in the study cohort, which may bias the patient population towards the inclusion of less healthy patients. In addition, a detailed evaluation of patients’ previous use of statins, including tolerance or history of adverse effects, was not conducted. These variables may explain lack of use of lipid-lowering therapy or failure to reach LDL-C targets in some patients. The lack of information regarding pretreatment cholesterol levels prevented an evaluation of the proportion of very-high-risk patients who had achieved a ≥50% reduction in LDL-C, but still had levels ≥1.8 mmol/L.

We conclude that there remains a considerable treatment gap with regard to lipid management among patients at high-risk and very-high-risk of CVD in primary care in Europe. This treatment gap could be closed by more rigorous calculation of global CVD risk in potentially at-risk patients, with prescription of statins for those at high-risk or very-high-risk, and treatment intensification until guideline-recommended total cholesterol and LDL-C levels are achieved. Improving lipid management in higher risk patients, primarily by optimizing the use of statin therapy, could significantly reduce the burden of CVD in Europe.
